# Similarities in the induction of the intracellular pathogen response in *Caenorhabditis elegans* and the type I interferon response in mammals

**DOI:** 10.1002/bies.202300097

**Published:** 2023-09-04

**Authors:** Vladimir Lažetić, Lakshmi E. Batachari, Alistair B. Russell, Emily R. Troemel

**Affiliations:** ^1^ School of Biological Sciences University of California, San Diego La Jolla California USA; ^2^ Department of Biological Sciences The George Washington University Washington DC USA

**Keywords:** *C. elegans*, development, epithelial immunity, interferon, proteasome, purine metabolism, RIG‐I‐like receptor

## Abstract

Although the type‐I interferon (IFN‐I) response is considered vertebrate‐specific, recent findings about the Intracellular Pathogen Response (IPR) in nematode *Caenorhabditis elegans* indicate that there are similarities between these two transcriptional immunological programs. The IPR is induced during infection with natural intracellular fungal and viral pathogens of the intestine and promotes resistance against these pathogens. Similarly, the IFN‐I response is induced by viruses and other intracellular pathogens and promotes resistance against infection. Whether the IPR and the IFN‐I response evolved in a divergent or convergent manner is an unanswered and exciting question, which could be addressed by further studies of immunity against intracellular pathogens in *C. elegans* and other simple host organisms. Here we highlight similar roles played by RIG‐I‐like receptors, purine metabolism enzymes, proteotoxic stressors, and transcription factors to induce the IPR and IFN‐I response, as well as the similar consequences of these defense programs on organismal development.

## Graphical abstract

This review provides a comparative analysis of the *Caenorhabiditis elegans* intracellular pathogen response and the mammalian type‐I interferon response. Both responses are transcriptional immunological programs that are regulated by similar features – including RIG‐I‐like signaling, perturbations to purine metabolism, proteotoxic stress, and transcription factors – to impact pathogen resistance and development.

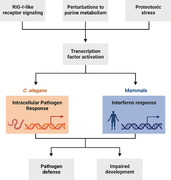

**Abbreviations**
ADA: adenosine deaminase
AGS: Aicardi–Goutieres syndrome
CARD: caspase activation and recruitment domain
cGAS: cyclic GMP–AMP synthase
CTD: C‐terminal domain
DAMP: damage‐associated molecular pattern
dsRNA: double‐stranded ribonucleic acid
ERV: endogenous retroviral element
GPCR: G‐protein‐coupled receptor
IFN: interferon
IPR: intracellular pathogen response
IRF: interferon regulatory factor
ISGF3: interferon‐stimulated gene factor 3
NHR: nuclear hormone receptor
ORR: oomycete recognition response
PAMP: pathogen‐associated molecular pattern
pDC: plasmacytoid dendritic cell
PNP: purine nucleoside phosphorylase
PRAAS: proteasome‐associated autoinflammatory syndrome
PRR: pattern recognition receptor
RLR: RIG‐I‐like receptors
RNAi: ribonucleic acid interference
siRNA: small interfering ribonucleic acid
SLE: systemic lupus erythematosus

## INTRODUCTION

Constant pressure from rapidly evolving pathogenic threats drives gene duplication, alteration and acquisition of new gene function, and even gene loss.^[^ ^1^ ^]^ These changes lead to complex patterns resulting from divergent and convergent evolution within immune pathways. We can see this complexity when comparing the nematode *Caenorhabditis elegans* to other metazoans. For example, *C. elegans* does not have the NF‐κB transcription factor, which is a key immune defense factor in the fruit fly *Drosophila melanogaster* and in vertebrates.^[^ ^2^ ^]^ On the other hand, RIG‐I‐like receptors (RLR) do not seem to be present in *D. melanogaster*, but they serve as important cytosolic viral RNA sensors in vertebrates and in *C. elegans*, where they are among the few pattern recognition receptors (PRRs) shared with vertebrates.^[^ ^3^ ^]^

RLRs promote anti‐viral defense in mammals by upregulating the transcription of type I interferon (IFN) genes, which encode secreted proteins that induce both local and systemic immune responses.^[^ ^4^ ^]^ In general, IFN responses represent some of the most important antiviral programs in all jawed vertebrates. Evolutionary studies indicate that the first IFN genes emerged in cartilaginous fish and were passed onto higher vertebrates.^[^ ^5^ ^]^ The IFN and interleukin‐10 (IL‐10) gene families evolved from the same ancestral gene, which likely encoded a helical cytokine. IFN‐I and IFN‐II genes probably separately diverged from the IL‐10 family in an early period of vertebrate evolution, whereas IFN‐III likely resulted from an IFN‐I duplication event in amphibians. While many of the topics we cover in this review relate to both IFN‐I and IFN‐III, for simplicity we will only cover IFN‐I responses.

**FIGURE 1:**
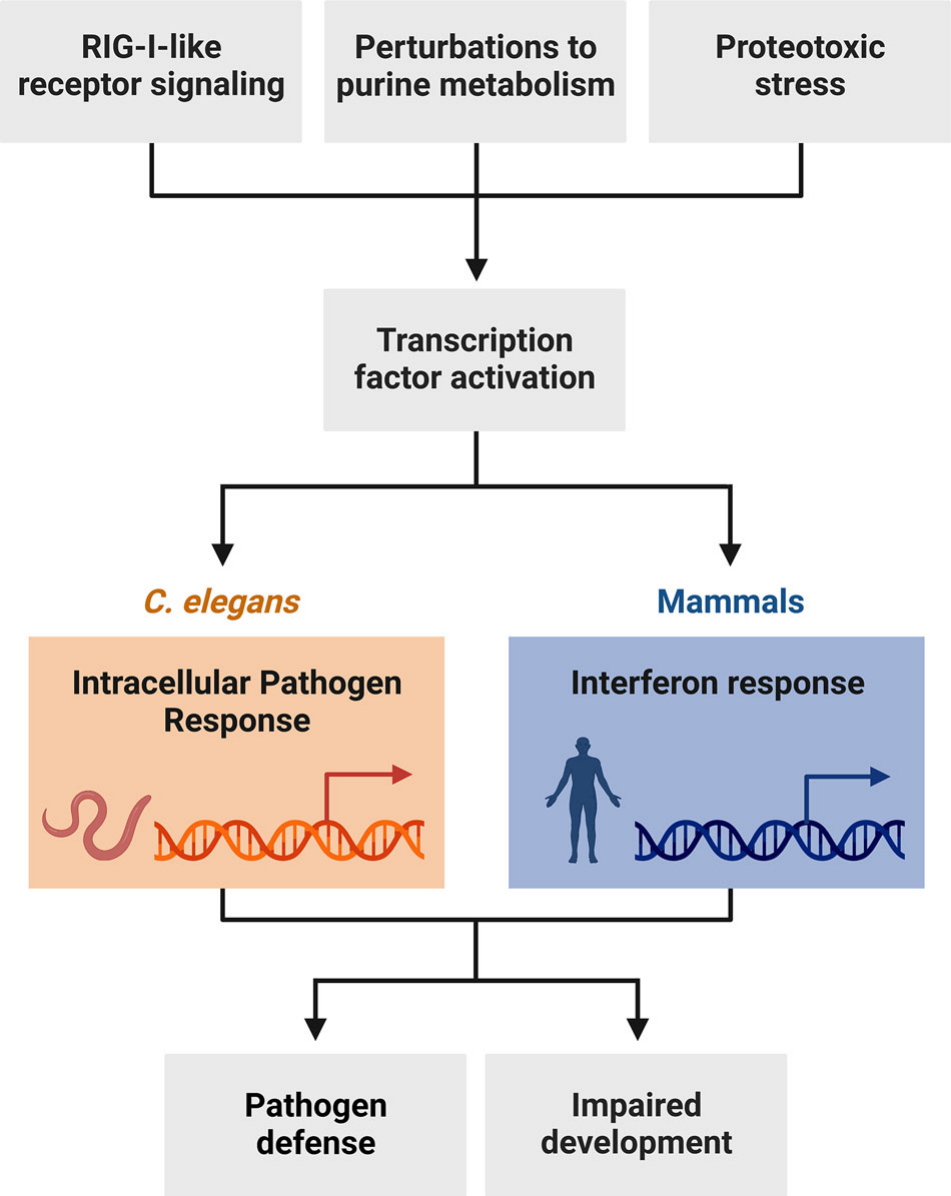
Overview of the similarities between the IPR in *C. elegans* and the IFN response in mammals. RIG‐I‐like receptors, perturbations of purine salvage/degradation metabolism, and proteotoxic stressors all activate transcription factors that trigger the IPR and IFN‐I responses, which promote pathogen defense and negatively affect development.

Given that IFN genes are vertebrate‐specific, how do RLRs in the invertebrate *C. elegans* promote anti‐viral immunity? Initial characterization indicated that RLRs promote defense through upregulating RNA interference (RNAi) in the nematode, with more recent work indicating that the RLR DRH‐1 also activates a transcriptional immune/stress response called the Intracellular Pathogen Response (IPR).^[^ ^3^, ^6^ ^]^ Thus, RLRs in both mammals and *C. elegans* are upstream of anti‐viral transcriptional responses. Furthermore, perturbations in purine salvage metabolism and proteostasis induce the IFN‐I response in mammals, as well as the IPR in *C. elegans*. A shared consequence of IFN‐I and IPR activation is impaired organismal development. Inspired by these and other similarities, this review will discuss the induction of these immune responses to intracellular pathogens of *C. elegans* and mammals, as well as the cost to host fitness incurred by their activation (Figure 1).

## *C. ELEGANS* HOST/PATHOGEN INTERACTIONS AND IMMUNE RESPONSES

*C. elegans* has a relatively simple anatomy, consisting of epithelial, neuronal, muscular, and germline tissues.^[^ ^7^ ^]^ So far, there has not been the identification of professional immune cells, like macrophages or neutrophils in worms.^[^ ^2^ ^]^ Thus, *C. elegans* heavily relies on non‐professional immune cells (i.e., cells whose primary function is not immunity‐related) like epithelial cells and neurons to defend against pathogens. Because of the many genetic tools that are available in *C. elegans* research, as well as the worm's transparent body and short generation time, *C. elegans* represents a powerful system for studying the defense mechanisms of non‐professional immune cells in a whole‐animal context.^[^ ^8^ ^]^

In nature, *C. elegans* lives in a microbially rich environment of rotting vegetation where it feeds on different types of microorganisms. Besides being a food source, many microbial species appear to form complex communities in the intestinal lumen of worms in the wild, and emerging research suggests that the gut microbiome has substantial effects on *C. elegans* development and pathogen resistance.^[^ ^9^, ^10^, ^11^ ^]^ In addition, *C. elegans* frequently encounters pathogenic microorganisms in its natural environment, including bacterial, viral, fungal, and oomycete pathogens.^[^ ^12^ ^]^

In a laboratory setting, synchronized populations of *C. elegans* are typically hatched as germ‐free organisms that are fed specific bacterial food sources (most commonly *Escherichia coli* strain OP50) and then challenged by specific pathogens. While infections with extracellular pathogens typically require exposure to pathogens as a sole food source, intracellular pathogens are most commonly provided in a mixture with standard *E. coli* food.^[^ ^13^ ^]^ This exposure to selected microbes allows researchers to directly study host‐pathogen interactions and their consequences. One of the best‐studied pathogens in *C. elegans* is the predominantly extracellular bacterial pathogen *Pseudomonas aeruginosa*. This microorganism produces several virulence factors that contribute to lethal intestinal infection in *C. elegans*, and studies with this and other predominantly extracellular pathogens have led to the discovery of various defense responses in worms.^[^ ^2^, ^14^ ^]^ For example, the upregulation of anti‐microbial genes via activation of a conserved p38 MAPK pathway provides cell‐autonomous defense against several pathogens of *C. elegans*.^[^ ^15^, ^16^, ^17^ ^]^ In addition, there are several examples of systemic regulation of immune responses whereby the nervous system controls defense in distal tissues like the intestine and epidermis.^[^ ^18^, ^19^, ^20^, ^21^, ^22^, ^23^ ^]^ For example, detection of epidermal pathogens called oomycetes by the nervous system leads to activation of the oomycete recognition response (ORR) in the epidermis.^[^ ^22^ ^]^

Despite many important discoveries regarding immune signaling and behavioral responses in *C. elegans*, only a few PRRs that recognize pathogen‐associated molecular patterns (PAMPs) and damage‐associated molecular patterns (DAMPs) have been identified in this host. With the exception of RLRs, *C. elegans* lacks obvious homologs of many of the canonical PRRs found in mammals, such as cyclic GMP–AMP synthase (cGAS)‐STING and nucleotide‐binding and leucine‐rich repeat (NLR) receptors. Interestingly, most of the few known PRRs in *C. elegans* belong to gene families that have expanded dramatically in *C. elegans* compared to mammals, such as the G‐protein‐coupled receptor (GPCR) family (estimates vary; up to 1,596 members in *C. elegans* and up to 948 in humans) and the nuclear hormone receptor (NHR) gene families (284 members in *C. elegans* compared to 48 in humans).^[^ ^24^, ^25^, ^26^ ^]^ For example, the GPCR DCAR‐1 can by activated either by infection of epidermal epithelial cells with the fungal pathogen *Drechmeria coniospora* or by physical wounding. DCAR‐1 serves as a DAMP receptor that recognizes the tyrosine‐derivative 4‐hydroxyphenyllactic acid in damaged tissue and induces an epidermal innate immune response.^[^ ^27^ ^]^ PCDR‐1 is another GPCR that may function as a PRR given its requirement for clearance of the bacterial pathogen *Microbacterium nematophilum* from the rectum.^[^ ^28^ ^]^ Recent characterization of NHR‐86 highlights its role as a novel type of PRR acting in intestinal epithelial cells. NHR‐86 binds to the newly described PAMP, *P. aeruginosa* metabolite phenazine‐1‐carboxamide, which activates an antibacterial transcriptional response in *C. elegans*.^[^ ^29^, ^30^ ^]^

Distinct pathogens elicit distinct transcriptional responses in *C. elegans*. However, the correlation between pathogen class and response type is less clear for *C. elegans* than it is for other hosts like *D. melanogaster*, where fungal and Gram‐positive bacterial pathogens elicit specific transcriptional responses through the Toll receptor, while Gram‐negative bacterial pathogens elicit distinct transcriptional responses through the Imd receptor.^[^ ^31^, ^32^, ^33^ ^]^ Interestingly, the IPR introduced above is a common transcriptional response in *C. elegans* induced by diverse intracellular pathogens of the intestine, including a natural RNA virus called the Orsay virus, and a species of microsporidia (fungal pathogens), called *Nematocida parisii*.^[^ ^34^, ^35^, ^36^, ^37^ ^]^ Though molecularly distinct, these microbes are both obligate intracellular pathogens that infect the *C. elegans* intestine in the wild. The IPR constitutes a novel immune/stress response that is mostly distinct from the responses elicited by facultative intracellular fungal pathogens and by bacterial pathogens like *P. aeruginosa*.^[^ ^34^, ^35^, ^36^, ^37^, ^38^, ^39^ ^]^ Notably however, the IPR has substantial overlap with the ORR, and these two transcriptional programs may represent distinct but related responses induced by pathogens of the intestine and the epidermis, respectively.^[^ ^22^, ^40^ ^]^

The IPR consists of about 80 highly upregulated genes, together with hundreds of additional genes upregulated at lower levels.^[^ ^34^, ^37^, ^38^ ^]^ Some IPR genes encode cullin‐RING ubiquitin ligase components, several of which are involved in defense against intracellular pathogens, as well as promoting proteostasis capacity, which is often perturbed by intracellular infection. IPR genes also include genes of unknown biochemical function called *pals* genes, which contain an uncharacterized ALS2CR12 signature named for its human homolog of unknown function.^[^ ^41^ ^]^ Interestingly, only one *pals* gene each has been identified in mice and humans, while the family has expanded to 39 members in *C. elegans*. Of the 39 genes, 26 are upregulated as part of the IPR, while several *pals* genes in *C. elegans* are not upregulated and instead serve as regulators of IPR gene transcription.^[^ ^34^, ^36^, ^37^, ^42^ ^]^ These *pals* genes appear to act in modules including activators and repressors. For example, *pals‐22* is a repressor of upregulated *pals* genes and all other IPR genes, and acts together with its antagonistic paralog *pals‐25*, functioning downstream of *pals‐22* as an activator of upregulated *pals* genes and other IPR genes in *pals‐22* mutants. *pals‐22* mutants exhibit constitutive expression of IPR genes, and thus display increased resistance to intracellular intestinal‐specific pathogens in a *pals‐25*‐dependent manner.^[^ ^34^, ^37^ ^]^

**FIGURE 2:**
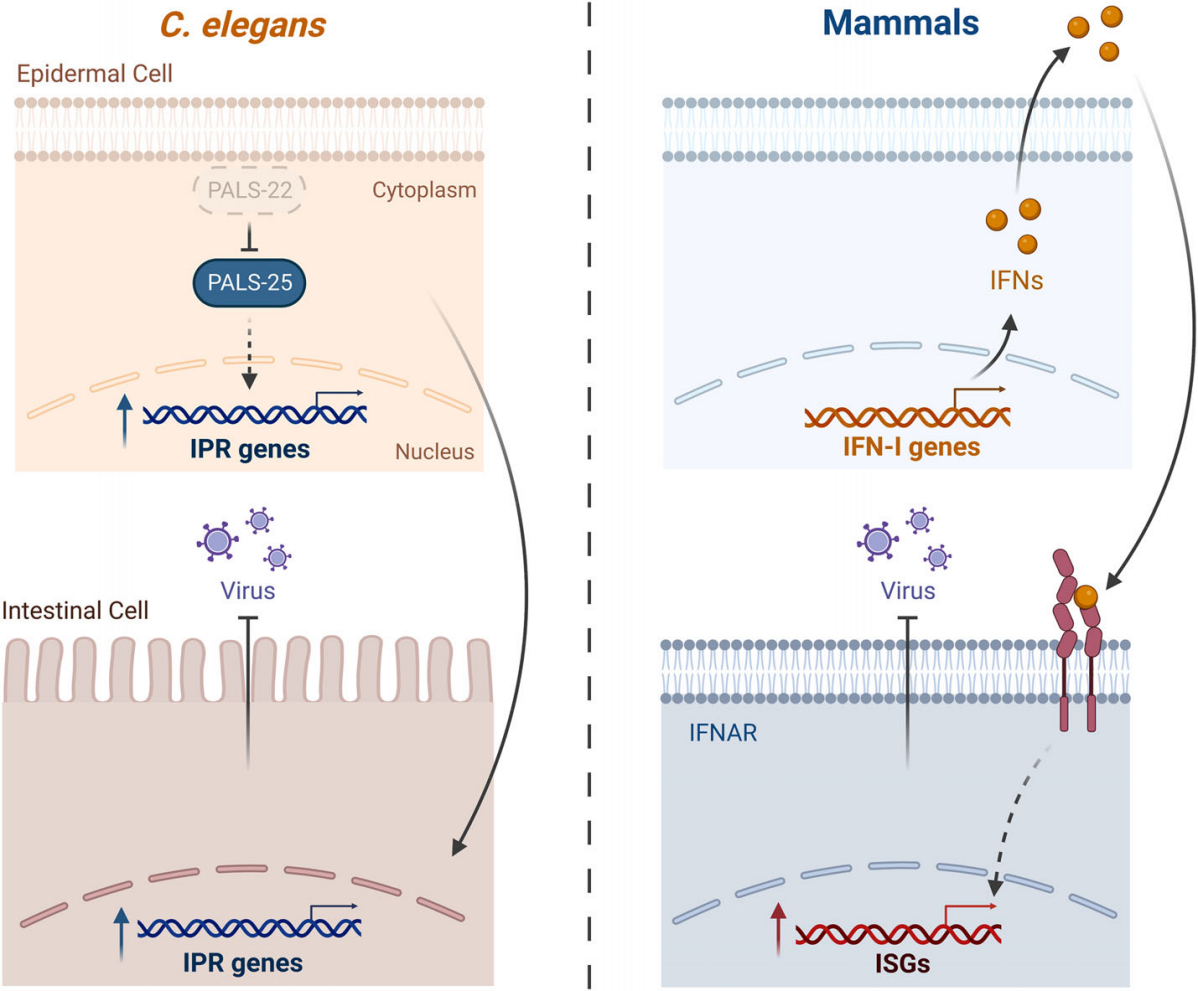
The IPR and IFN response enhance immunity via cell non‐autonomous signaling. Depletion of PALS‐22 protein in epidermal cells leads to activation of the IPR in both epidermal and intestinal cells, as well as increased resistance against intracellular intestinal pathogens. Secreted type‐I interferons bind the interferon‐α/β receptor (IFNAR) to induce interferon‐stimulated genes (ISGs) and antiviral immunity in other cell types.

Because *pals‐22* and *pals‐25* are broadly expressed in virtually all tissues of *C. elegans*, these regulators may act as an ON/OFF switch to coordinate immunity in several different tissues. Indeed, independent genetic screens identified *pals‐22* and *pals‐25* as regulators of the ORR induced by oomycetes, which are epidermal pathogens, as well as the IPR induced by viral and microsporidian intestinal pathogens.^[^ ^34^, ^36^, ^37^ ^]^ In mammals, coordinated signaling across cells and tissues is a key feature of the IFN‐I response. The response starts with infected cells or proximal bystander cells upregulating transcription of IFN‐I genes, which encode proteins that are secreted extracellularly, where they bind to the IFN‐I receptor expressed on other cells.^[^ ^43^ ^]^ These activated cells then trigger the expression of a large suite of interferon‐stimulated genes (ISGs) (Figure 2).^[^ ^44^, ^45^, ^46^, ^47^ ^]^ Because the IPR is still being defined in *C. elegans*, it is not yet clear which IPR genes are induced as part of the initial transcriptional response (potentially analogous to IFN‐I in mammals), and which genes are induced as part of a secondary response in uninfected cells (potentially analogous to ISGs in mammals). It is also possible that the IPR pathway may not have a two‐step structure like the IFN‐I response. It is also unclear which IPR components may act as IFNs. Nonetheless, coordination of the IPR across cells and tissues in *C. elegans* suggests that there are likely secreted signaling proteins that activate this response in distal tissues.^[^ ^34^ ^]^ In particular, activation of the IPR through depletion of PALS‐22 protein in epidermal cells, or through the epidermal‐specific expression of a gain‐of‐function form of PALS‐25 protein, triggers IPR activation in epidermal cells as well as in intestinal epithelial cells (Figure 2). Furthermore, this epidermal‐specific activation of the IPR leads to increased resistance to intracellular infection of the intestine.^[^ ^34^ ^]^ While much is still to be learned about how this response is coordinated from the epidermis to the intestine, these findings indicate that the IPR, like the IFN‐I response, regulates systemic immunity against intracellular pathogens.

## RIG‐I‐LIKE RECEPTORS (RLRs) REGULATE THE IFN‐I RESPONSES AND THE IPR

**FIGURE 3:**
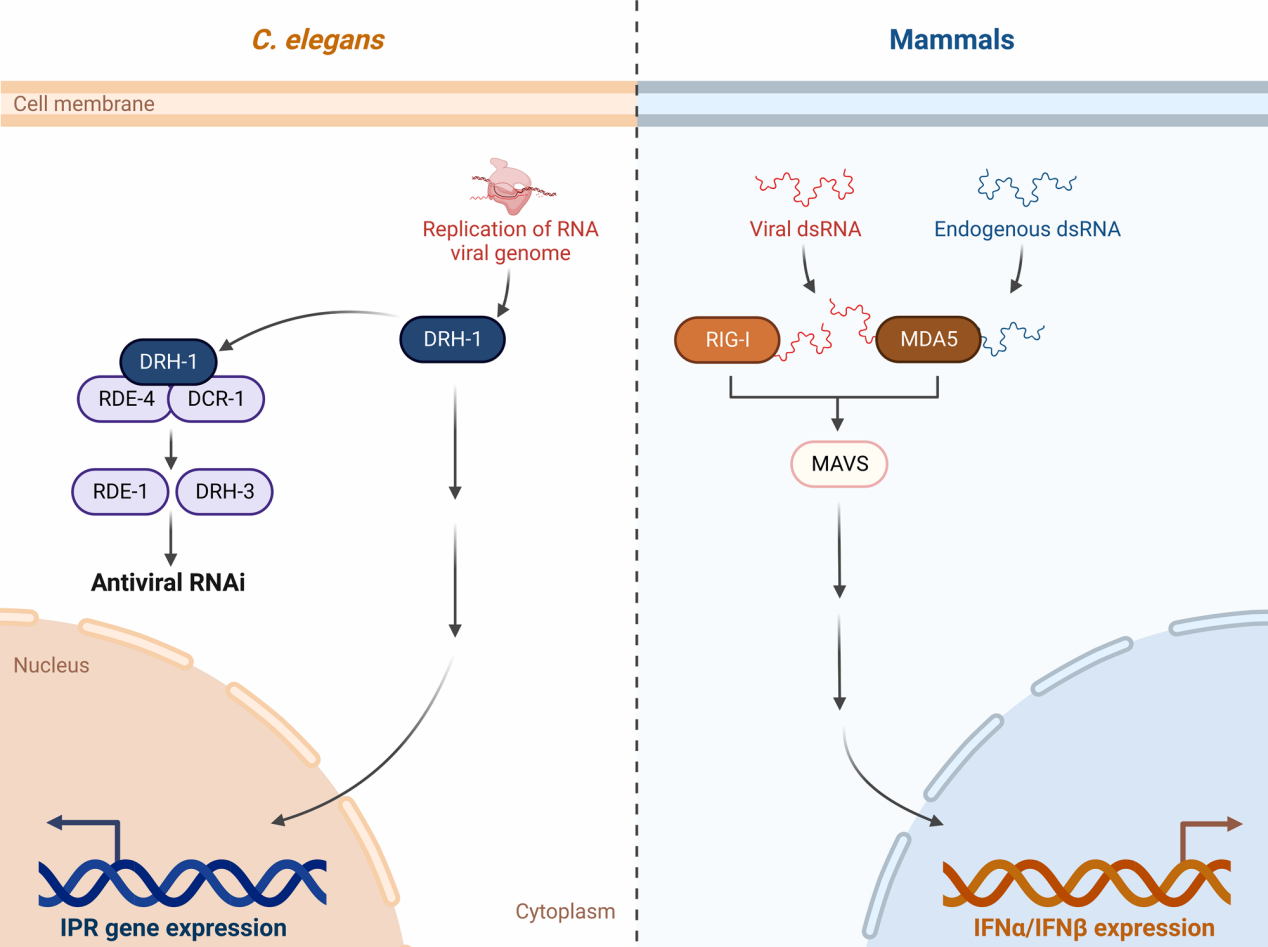
Activation of the IPR and IFN response through the RIG‐I‐like receptors during viral infection. *C. elegans* RIG‐I‐like protein DRH‐1 activates the IPR following viral genome replication. DRH‐1 is also a component of the RNAi complex. Mammalian RIG‐I and MDA5 recognize viral dsRNA and signal through MAVS to activate downstream factors that induce transcription of IFN genes.

RLRs and their homologs play central roles in antiviral defense across diverse metazoan lineages. Phylogenetic analysis suggests that RLRs emerged at the inception of multicellularity in metazoans, as RLRs are not found in non‐metazoan eukaryotes or plants.^[^ ^48^ ^]^ Gene duplication led to functional heterogeneity of RLRs across different species.^[^ ^48^ ^]^ The *C. elegans* genome contains three mammalian RLR homologs, known as dicer‐related helicase 1, 2, and 3 (*drh‐1*, *drh‐2*, and *drh‐3*).^[^ ^6^, ^49^ ^]^ The gene duplication events that led to the three *C. elegans* RLR homologs occurred independently from duplication events that led to mammalian RLR homologs. As such, we might expect significant divergence in the function of *C. elegans* RLR homologs compared to homologs found in other organisms. Indeed, several studies indicate that DRH‐1 regulates RNAi, a function distinct from RLRs in mammals, which activate transcription of the ligands IFN‐α/β involved in the IFN‐I response (Figure 3).^[^ ^6^, ^49^ ^]^ Protein interaction analysis indicated that DRH‐1 binds two key RNAi components in *C. elegans*, the RNase III‐related enzyme Dicer (DCR‐1) and the RNA helicase RDE‐4, to form a complex that initiates RNAi in response to exogenous double‐stranded RNA (dsRNA). Because dsRNA is a product of viral replication, these findings suggested that DRH‐1 could function in the detection of RNA virus infection. These and other findings indicated that DRH‐1 regulates the processing of dsRNA to small interfering RNAs (siRNAs), while another Dicer‐interacting protein, DRH‐3, mediates the production of secondary siRNAs and is required for RNAi in the germline.^[^ ^49^ ^]^

Following the initial identification of DRH‐1 and its role in RNAi, subsequent genetic analysis indicated that DRH‐1 inhibits replication of a heterologously expressed Flock house virus replicon. This anti‐viral effect differs from the previously described RNAi‐mediated silencing triggered by non‐viral exogenous dsRNA.^[^ ^50^ ^]^ The same study suggests that *drh‐2* may be a negative regulator of antiviral RNAi. In 2011, the discovery of the Orsay virus from wild *C. elegans* was significant, as it allowed studies for the first time with a virus that can infect worms via feeding instead of using artificial delivery methods, and which can complete its life cycle in *C. elegans* in the lab.^[^ ^51^ ^]^ Studies with the Orsay virus also demonstrated a clear role for RNAi in antiviral defense.^[^ ^51^ ^]^ Excitingly, analysis of wild *C. elegans* strains that had differing levels of resistance to the Orsay virus revealed that this difference was due to a naturally occurring deletion polymorphism in *drh‐1* that causes increased sensitivity to infection.^[^ ^52^ ^]^ Further characterization revealed that *drh‐1* mutants exhibit defects in antiviral RNAi to the Orsay virus as well as to vesicular stomatitis virus, which is introduced into *C. elegans* via microinjection.^[^ ^52^, ^53^, ^54^ ^]^ However, the specific function of DRH‐1 in the RNAi pathway is unclear, as one study proposed that it is not required to initiate anti‐viral siRNA, but rather acts in a downstream step to enhance it.^[^ ^53^ ^]^ Regardless, DRH‐1 does appear to be important for promoting anti‐viral RNAi against several viruses and to protect against infection. Interestingly, while *D. melanogaster* lacks an obvious RLR homolog, it does have an RNA helicase gene called Dicer2, which is important for anti‐viral RNAi in this species.^[^ ^55^, ^56^ ^]^

Recent work indicates that *C. elegans* RLRs may have an additional anti‐viral role similar to mammalian RLRs. As mentioned above, during infection with the Orsay virus, DRH‐1 is essential for the activation of the transcription of IPR genes. Moreover, the requirement for DRH‐1 appears to be specific to viral infection, as DRH‐1 is dispensable for IPR activation in response to non‐viral triggers, such as microsporidia and proteotoxic stress.^[^ ^3^ ^]^ DRH‐1 also activates the IPR in a manner independent of canonical RNAi components including DCR‐1/Dicer, and RNA‐binding proteins RDE‐4 and RDE‐1, suggesting that DRH‐1 upregulates transcription in a manner distinct from its effects on RNAi (Figure 3).^[^ ^3^ ^]^

In mammals, members of the RLR family include the founding member retinoic acid‐inducible gene I (RIG‐I), melanoma differentiation‐associated gene 5 (MDA5), and laboratory of genetics and physiology 2 (LGP2).^[^ ^4^, ^57^, ^58^ ^]^ In the canonical signaling pathway, mammalian RLRs detect viral or host‐derived RNAs to initiate a series of downstream signaling events that result in the transcription of IFN genes (Figure 3).^[^ ^59^ ^]^ All RLRs share a domain architecture composed of a central DExD/H box RNA helicase domain and a C‐terminal domain (CTD). RIG‐I and MDA5 also possess N‐terminal caspase activation and recruitment domains (CARDs) that mediate interactions with downstream host signaling factors. On the other hand, LGP2 lacks N‐terminal CARDs and does not seem to activate the IFN response. The role of LGP2 is potentially quite varied, as some reports suggest that LGP2 is a positive regulator of RLR signaling, whereas others describe LGP2 as a negative regulator.^[^ ^60^, ^61^, ^62^ ^]^ Nevertheless, a shared characteristic of all three mammalian RLRs (RIG‐I, MDA5, and LGP2) is the ability to bind RNA at the helicase and CTD.

Upon binding RNA, mammalian RIG‐I and MDA5 adopt a signaling‐competent configuration. Mammalian RIG‐I/MDA5 bind RNA virus replication products, including 5′ triphosphate RNA and dsRNA.^[^ ^63^ ^]^ In addition to binding RNAs of viral origin, RIG‐I and MDA5 also bind host RNAs (Figure 3).^[^ ^64^ ^]^ For example, perturbations to mitochondrial homeostasis can induce the release of mitochondrial dsRNA, which is then recognized by MDA5 to induce an IFN‐I response.^[^ ^65^ ^]^ While these and other studies have elucidated the RNA‐binding function of mammalian RLRs, little is known about whether RNA‐binding is a broadly conserved function in RLRs from other species.

Several studies suggest that *C. elegans* DRH‐1 may bind RNA. Specifically, studies mentioned above showed that DRH‐1 interacts with RNA‐binding proteins.^[^ ^6^ ^]^ In addition, more recent findings with a genetically encoded viral replicon demonstrated that replication products of the Orsay virus (presumably including dsRNA) induce the majority of IPR genes in a DRH‐1‐dependent manner (Figure 3).^[^ ^3^, ^66^ ^]^ Although direct binding of RNA to DRH‐1 has not been demonstrated, these observations support the idea that DRH‐1 recognizes a viral intermediate to induce a transcriptional response, analogous to RLR sensing of viral replication products. Further, the helicase and C‐terminal domains of DRH‐1 share relatively high sequence similarity and conserved amino acid motifs with human RLRs. The human RIG‐I helicase domain and CTD can even functionally substitute for corresponding domains in DRH‐1 in the context of antiviral RNAi.^[^ ^67^ ^]^ In addition, mitochondrial dysfunction in *C. elegans* induces RNAi via DRH‐1, and is associated with upregulation of mitochondrial RNA, in a situation that is perhaps analogous to RLR binding mitochondrial dsRNA in mammals.^[^ ^68^ ^]^ Collectively, these findings support the idea that RNA‐binding activity is conserved in DRH‐1/RLR and that interaction between viral RNA and DRH‐1 may regulate activation of the IPR, similar to what is observed in the mammalian antiviral IFN‐I response.

Sequence and functional analyses of DRH‐1 have unveiled similarities in the helicase and C‐terminal domain with RIG‐I/MDA5, whereas the N‐terminal domain appears to be more divergent.^[^ ^52^ ^]^ RIG‐I/MDA5 CARDs interact with the CARD domain in mitochondrial antiviral signaling protein (MAVS), and nucleate a signaling complex resulting in transcription of IFN‐α/β (Figure 3). In *C. elegans*, it remains unclear if DRH‐1 contains N‐terminal CARDs that mediate signaling to the IPR, because the N‐terminus of RIG‐I/MDA5 shares low amino acid sequence identity with the N‐terminus of DRH‐1. One possibility is that the protein structure, and thus signaling activity, is conserved despite disparate sequences. Alternatively, the sequence divergence at the N‐terminus of DRH‐1 could reflect a novel mode of RLR signaling that does not involve CARD domains. In line with the possibility of a novel mode of signaling, *C. elegans* lacks obvious homologs to the downstream signaling components of the RLR pathway, including MAVS. Further investigation of how DRH‐1 regulates an antiviral transcriptional response in *C. elegans* could advance our understanding of how RLRs activate anti‐viral defense apart from classic IFN‐I responses, and how these responses have been conserved or rewired in metazoans.

## THE ROLE OF NUCLEOTIDE METABOLISM IN REGULATING IMMUNOLOGICAL RESPONSES

In addition to the IPR and the IFN‐I response both being regulated by RLRs, these two responses are also regulated by similar enzymes involved in nucleotide metabolism. Nucleotides are widely known as the fundamental units of DNA and RNA, but nucleotides and nucleotide metabolism also play vital roles in modulating host immune responses during viral infection. For example, there are numerous examples of cyclic di‐nucleotides serving as second messengers in cell‐intrinsic immune signaling pathways.^[^ ^69^, ^70^ ^]^ Furthermore, there are many host antiviral factors that deplete or modify nucleotides to block viral replication.^[^ ^71^, ^72^ ^]^ The abundance of purine nucleotides in particular represents a limiting step in viral replication for two reasons: (1) purines like adenine and guanine are indispensable constituents of rapidly amplifying viral genomes, and (2) they represent the core components of nucleoside triphosphates, which comprise the main energy sources in the cell. Interestingly, microsporidia genomes lack nucleotide biosynthesis pathways, and so these obligate intracellular pathogens likely depend entirely on hosts for purines and other nucleotides, similar to viruses.^[^ ^39^, ^73^, ^74^, ^75^ ^]^ As microsporidia are enclosed in membranes that separate them from the host cytoplasm, they express nucleotide transporters on their plasma membranes that ‘steal’ purines from the host cytoplasm.^[^ ^73^, ^74^ ^]^

Cells synthesize purine nucleotides through either the *de novo* or the salvage pathways, with a preference for the more energy‐efficient salvage pathway.^[^ ^39^, ^76^ ^]^ The enzymes in purine salvage pathways are highly conserved from bacteria to humans.^[^ ^77^ ^]^ Two key enzymes in the purine nucleotide salvage pathway are adenosine deaminase (ADA) and purine nucleoside phosphorylase (PNP). ADA catalyzes the deamination of (deoxy‐) adenosine to (deoxy‐) inosine, whereas PNP mediates cleavage of the N‐glycosidic bond in (deoxy‐) inosine and (deoxy‐) guanosine to hypoxanthine and guanine, respectively. Humans have two ADA enzymes: ADA2 has a signal sequence and is responsible for most of the extracellular activity, while ADA1 lacks a signal sequence and is responsible for most of the intracellular activity.^[^ ^78^, ^79^ ^]^ Mutations in ADA1, ADA2, and PNP salvage enzymes lead to complex syndromes in humans that include features of immunodeficiencies coupled with auto‐inflammation.^[^ ^80^, ^81^, ^82^ ^]^

Of relevance for this review is the finding that loss of ADA2 leads to increased IFN‐β mRNA expression and spontaneous IFN‐I signaling in human endothelial cells, providing insight into the potential basis for diseases caused by loss of ADA2.^[^ ^83^ ^]^ The mechanism proposed by Dhawani et al is that loss of ADA2 leads to increased extracellular deoxy‐adenosine that is taken up into cells and converted into deoxy‐inosine by intracellular ADA1. Increased intracellular deoxy‐inosine then blocks SAM synthetase activity, leading to hypomethylation on DNA of endogenous retroviral elements (ERVs). Given that methylation normally silences these ERVs, hypomethylation causes increased transcription, which is bidirectional, generating ERV dsRNA. This dsRNA then activates RLRs, triggering an IFN‐I response and antiviral immunity.^[^ ^83^ ^]^ The effect of ADA2 loss can be exacerbated by loss of PNP, likely due to a further increase in deoxy‐inosine, although PNP loss in a wild‐type background was not described in this study. Altogether, these results provide a mechanism by which dysregulated purine metabolism, through the loss of ADA2 in human endothelial cells, triggers the IFN‐I response. Of note, human epithelial cells do not appear to express ADA2, and future work could explore whether ADA1 (and also PNP) have a role in regulating IFN‐I in these cells.

Analogous to how mutations in purine salvage enzymes lead to upregulated IFN‐I responses in human cells, perturbations in a purine salvage enzyme function also result in the activation of the IPR. A forward genetic screen in *C. elegans* uncovered the purine nucleoside phosphorylase‐encoding gene *pnp‐1* as a negative regulator of the IPR. Loss of *pnp‐1* leads to upregulation of the IPR gene expression and provides resistance to Orsay virus and microsporidia infections.^[^ ^35^, ^39^ ^]^ Metabolomic analysis showed that *pnp‐1* mutants have increased levels of the PNP substrate inosine, as well as decreased levels of the PNP product, hypoxanthine. This result confirmed that the PNP‐1 protein in *C. elegans* has an enzymatic activity similar to PNP in other species. As previously stated, the accumulation of deoxy‐inosine in mammalian cells induces the IFN response through ERV upregulation. However, it is currently unknown if increased inosine concentrations in worms induce the IPR through a similar mechanism, although the *C. elegans* genome does contain retroviral‐like sequences.^[^ ^84^ ^]^ While *pnp‐1* is expressed in neurons and intestinal epithelial cells, expression of *pnp‐1* specifically in the intestine was sufficient to rescue *pnp‐1* mutant phenotypes, indicating intestinal epithelial cells are the site of action for inducing immune responses due to loss of *pnp‐1*.^[^ ^39^ ^]^ Taken together, these studies indicate that purine salvage metabolism plays an important function in regulating innate immune responses in both mammals and nematodes. Future studies could investigate the role of *adah‐1*, the *C. elegans* homolog of ADA1/2, and the mechanisms by which alterations in purine salvage metabolism induce immune responses in this host in comparison to humans.

## PROTEOTOXIC STRESS AS A REGULATOR OF THE IFN‐I RESPONSE AND THE IPR

Another area of similarity between the IFN‐I response and the IPR is that they both can be triggered by stressors that impair protein homeostasis (proteostasis). Obligate intracellular pathogens commonly perturb proteostasis, as they undergo their entire replicative life cycle inside host cells.^[^ ^85^ ^]^ In particular, viruses cause proteotoxic stress by hijacking host protein synthesis machinery to synthesize viral proteins, thus impairing the synthesis of host proteins. And while microsporidia presumably synthesize their own proteins, studies indicate that they secrete hundreds of proteins into the host cytosol, which likely impair proteostasis in the host cell.^[^ ^86^ ^]^ Indeed, infection of *C. elegans* by either the Orsay virus or *N. parisii* leads to the formation of ubiquitin aggregates, which are hallmarks of impaired proteostasis.^[^ ^38^ ^]^

Perturbations in proteostasis by non‐pathogenic triggers in *C. elegans* can also activate the IPR, as inhibition of the proteasome either genetically or pharmacologically leads to IPR activation, as does knock‐down of ubiquitin expression or prolonged heat stress.^[^ ^38^ ^]^ RNA‐seq analysis revealed that IPR induction in response to proteasome blockade occurs in parallel to a previously described transcriptional response to proteasome blockade called the bounce‐back response.^[^ ^35^, ^37^, ^87^ ^]^ The bounce‐back response includes the upregulation of proteasome subunit genes, which are not part of the IPR. The bounce‐back response is controlled by SKN‐1, which is a transcription factor that promotes resistance to proteotoxic stress, oxidative stress, as well as pathogen infection.^[^ ^88^, ^89^, ^90^ ^]^ Overall, these findings in *C. elegans* suggest that proteasome blockade stimulates the activation of the IPR and the bounce‐back response as distinct, non‐overlapping stress/immune responses.

Studies in mammals indicate that blockade of the proteasome also induces two transcriptional responses with similarity to the responses in *C. elegans*. First, Nrf1, a transcription factor homologous to SKN‐1A, also upregulates the expression of proteasome subunits in response to proteasome blockade in this host.^[^ ^91^ ^]^ In the second response, blockade of the proteasome induces the IFN‐I response,^[^ ^92^ ^]^ similar to how it induces the IPR in worms. Moreover, mutations in the proteasome are associated with inflammatory disorders called interferonopathies that are characterized by overexpression of IFN‐I. Mutations in the gene encoding inducible proteasome (immunoproteasome) subunit β 8 (PSMB8) were reported to be related to the development of proteasome‐associated autoinflammatory syndrome (PRAAS) in humans.^[^ ^93^, ^94^, ^95^, ^96^ ^]^ Subsequently, mutations in four other genes that encode proteasome components were also found to cause PRAAS interferonopathy, including immunoproteasome and constitutive proteasome subunits.^[^ ^93^, ^97^ ^]^ Furthermore, PRAAS‐associated mutations have been identified in genes encoding several proteasome regulators.^[^ ^93^, ^97^ ^]^ Importantly, these mutants show increased expression of IFN genes and ISGs, which is likely a prerequisite for PRASS development.^[^ ^93^, ^98^ ^]^ These examples demonstrate a correlation between proteasomal dysfunction and innate immune induction and suggest that there are two conserved surveillance systems (SKN‐1A/Nrf1 and IPR/IFN‐I) that trigger protective transcriptional responses following proteostasis perturbations in both mammals and in *C. elegans*.

## TRANSCRIPTION FACTOR REGULATION OF IFN‐I GENE EXPRESSION AND THE IPR

**FIGURE 4:**
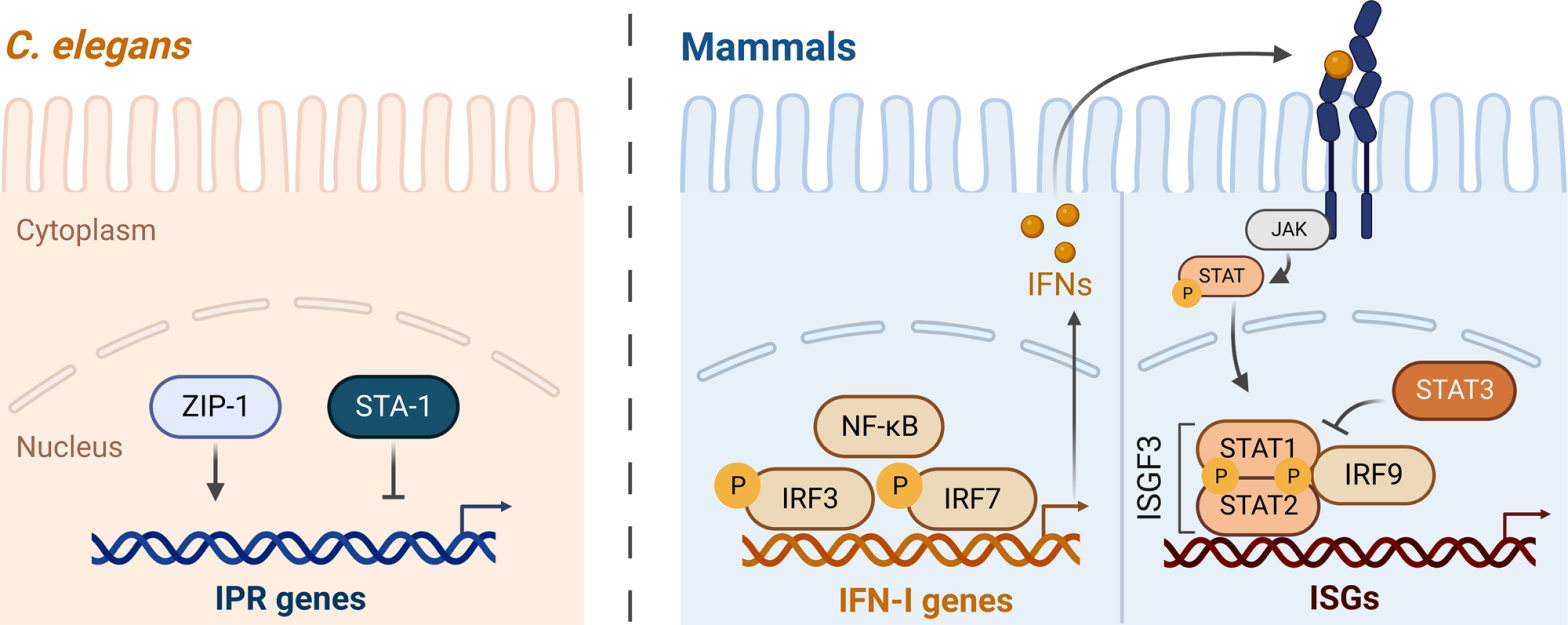
Overview of the transcriptional regulation IPR and IFN‐I responses, including intercellular signaling for IFN‐I response (while intercellular signaling does occur in the IPR, the exact players are not well‐understood). ZIP‐1 and STA‐1 antagonistically regulate the transcription of some IPR genes. NF‐κB and IRFs promote the transcription of IFN genes. IFN signals to the same (autocrine signaling) and other cells (paracrine signaling) by binding to JAK receptors on the cell surface. This binding stimulates the formation of the ISGF3 complex that consists of STAT1, STAT2, and IRF9 and activates the transcription of ISGs. STAT3 inhibits the ISGF3 complex. P = phosphorylated.

Having described the similarities in the activation of the IFN‐I response and the IPR, we now compare the transcription factors that activate these responses. The IFN‐I response is transcriptionally regulated by the members of the IFN regulatory factor (IRF) family. For example, IRF3 and IRF7 are essential for the activation of IFN‐I downstream of PRRs like RLRs (Figure 4).^[^ ^99^, ^100^ ^]^ IRF3 is ubiquitously expressed in all cell types, whereas IRF7 is highly expressed in dendritic cells. IRF7 is expressed in other types of cells as well, but its half‐life is relatively short in comparison to IRF3. IRF7 degradation via ubiquitin‐proteasome system is additionally accelerated upon viral infection in all tested cell types, except for plasmacytoid dendritic cells (pDCs). Following primary infection, pDCs are the most potent producers of IFN‐I, which likely correlates with the longer stability of IRF7 in this cell type.^[^ ^101^ ^]^ Although IRF7 degradation in pDCs is lower than in other cells, it still needs to be adequately regulated to prevent harmful IFN hyperactivation. This regulation is achieved through the polyubiquitylation and proteasomal degradation of IRF7.^[^ ^102^ ^]^ Polyubiquitylation also plays an important role in IRF3 turnover during viral infection.^[^ ^103^ ^]^ In the absence of pathogens, IRFs are maintained in their inactive form in the cytosol. Upon infection, IRFs become activated through phosphorylation, which leads to their translocation into the nucleus and transcriptional upregulation of IFN gene expression (Figure 4).

IFNs bind to the receptors on the plasma membranes of the target cells. The binding of IFNs leads to the activation of several JAK/STAT pathways that promote resistance to pathogens.^[^ ^104^ ^]^ For example, IFN‐I activates STAT1, STAT2, and STAT3 proteins, which regulate transcription. STAT1, STAT2, and IRF9 form the IFN‐stimulated gene factor 3 (ISGF3) complex that promotes the transcription of IFN‐stimulated genes, which are important for the antiviral response (Figure 4).^[^ ^105^ ^]^ However, STAT3 acts as a suppressor of IFN‐I signaling and prevents hyperactivation of the ISGF3 complex in several ways. For example, STAT3 prevents the transcription of STAT1, STAT2, and IRF9; it binds and sequesters STAT1, and it cooperates with PLSCR2 to prevent DNA binding of ISGF3. Furthermore, STAT3 indirectly suppresses ISGF3 through the induction of miRNAs that target this complex for degradation and through the induction of IFN‐negative regulator SOCS3.^[^ ^105^ ^]^ In summary, several STAT proteins play antagonistic roles to fine‐tune the antiviral response.

The *C. elegans* genome does not appear to contain homologs of mammalian IRFs, but it contains two genes that encode STAT family transcription factors STA‐1 and STA‐2, which are both important for innate immunity. STA‐1 is a negative regulator of the IPR (Figure 4) and *sta‐1* depletion leads to increased resistance to Orsay virus infection.^[^ ^106^ ^]^ This result suggests that STA‐1 in *C. elegans* and STAT3 in mammals might have similar roles as negative regulators of anti‐viral defense. STA‐2 promotes the expression of antimicrobial peptide genes in the epidermis and provides protection against the fungal pathogen *D. coniospora*.^[^ ^107^ ^]^ However, STA‐2 is not required for promoting the IPR and has no known role there.^[^ ^35^ ^]^

A recent study has identified ZIP‐1 as the first transcription factor that activates the IPR, and the first transcription factor shown to promote defense against the Orsay virus and microsporidia infection in *C. elegans* (Figure 4).^[^ ^35^ ^]^ ZIP‐1 acts as a central hub for all known IPR triggers and is required for the upregulation of a subset of IPR genes. ZIP‐1 belongs to the extended family of bZIP transcription factors that play important roles in plant immunity, as well as in antibacterial defense in nematodes, including bZIP transcription factors ZIP‐2 and ATF7 that provide defense against *P. aeruginosa* infection.^[^ ^108^, ^109^ ^]^ *zip‐1* mutants show significantly higher viral loads and have a shorter lifespan upon *N. parisii* infection in comparison to wild‐type control animals.^[^ ^35^ ^]^

RNA‐seq studies suggest that ZIP‐1 regulates the transcription of roughly one‐third of all IPR genes. While some IPR genes are *zip‐1*‐dependent only in the initial phase of IPR activation, others are dependent in the later phase. Therefore, IPR genes can be categorized into three groups: early *zip‐1*‐dependent, late *zip‐1*‐dependent, and *zip‐1*‐independent genes.^[^ ^35^ ^]^ Other, currently unknown transcription factors are necessary to induce the expression of *zip‐1*‐independent and partially *zip‐1*‐dependent genes. Tissue‐specific depletion studies of *zip‐1* indicate it acts in the intestine to regulate gene expression, although it is also expressed in epidermal cells, and may also have roles there.^[^ ^35^ ^]^

*zip‐1* itself is transcriptionally upregulated by IPR triggers. However, because it is required for mRNA induction only 30 min after treatment with an IPR trigger, it is believed to be the proximal transcription factor downstream of RLR activation and other IPR triggers, although it is not yet known where ZIP‐1 binds in the genome.^[^ ^35^ ^]^ And while *zip‐1* mRNA is present in the absence of IPR triggers, ZIP‐1::GFP protein reporter expression is not detectable without IPR activation, suggesting that ZIP‐1 protein may have a fast turnover similar to IRF7 in mammals. Following IPR activation, ZIP‐1::GFP expression becomes visible and is localized to the nucleus of infected and neighboring uninfected cells, similar to paracrine signaling during the IFN response.^[^ ^35^ ^]^ However, even before becoming visible by fluorescence microscopy, ZIP‐1 is required for induction of a subset of IPR genes. This result suggests that even when ZIP‐1 is expressed at very low levels, it still plays a key role for induction of immunological response following pathogen invasion.

## PROLONGED ACTIVATION OF THE IFN‐I RESPONSE AND THE IPR IS DETRIMENTAL TO ORGANISMAL DEVELOPMENT

Activation of immunological responses rewires metabolism to promote defense at the expense of cellular and organismal development and growth. Therefore, prolonged and/or overly strong activation of immune responses can be detrimental to organismal development and can cause metabolic diseases. For example, autoimmune disorders Aicardi–Goutieres syndrome (AGS) and systemic lupus erythematosus (SLE) show a correlation between hyperactivation of IFN‐I response and neurological developmental impairments. Specifically, AGS patients frequently develop cerebral atrophy and microcephaly,^[^ ^110^ ^]^ whereas chronic inflammation of different organ systems during SLE often leads to cellular necrosis and organ degeneration.^[^ ^111^, ^112^ ^]^ Loss of a recently described “guard” of the IFN‐I response called MORC3 leads to increased IFN‐I expression and resistance to viral infection, and mice with MORC3 mutations have bone and hematopoietic abnormalities.^[^ ^113^ ^]^ Enteroviral infections and a subsequent increase of IFN‐I signaling have been implicated in the autoimmune destruction of pancreatic cells and the onset of type 1 diabetes mellitus.^[^ ^114^, ^115^ ^]^ Diabetes and other metabolic disorders frequently have deleterious effects on organismal development.

Similar to hyperactivation of the IFN‐I response, prolonged activation of the IPR in *C. elegans* appears to be detrimental to development, reproduction and lifespan, based on analysis of loss‐of‐function mutations in two negative regulators of the IPR, *pals‐22* and *pals‐17*.^[^ ^36^, ^37^, ^42^ ^]^ Mutations in *pals‐22* or *pals‐17* cause constitutive upregulation of IPR gene expression in the absence of infection, and these mutants have increased resistance to intracellular pathogens as well as developmental delay. Specifically, *pals‐22* premature stop codon mutants develop slower than wild‐type animals, and have a slenderer appearance, smaller brood sizes, and shorter lifespan.^[^ ^36^, ^37^ ^]^ Furthermore, they exhibit phenotypes reminiscent of premature aging, including locomotory defects.^[^ ^41^ ^]^ Loss of *pals‐17* causes even more severe negative impacts than loss *pals‐22*. *pals‐17* partial loss‐of‐function mutants grow very slowly and asynchronously within a population, while deletion of the entire *pals‐17* gene causes arrested development of all animals at an early larval stage.^[^ ^42^ ^]^ Several other *pals* genes act downstream of *pals‐22* and *pals‐17* and antagonize their functions. Loss of *pals‐25* and *pals‐16* revert all phenotypes observed in *pals‐22* and *pals‐17* mutants, respectively.^[^ ^37^, ^42^ ^]^ Loss of the *pals‐20* gene, however, suppresses the upregulation of IPR genes in *pals‐17* mutants only at early larval stages and provides nearly normal development.^[^ ^42^ ^]^ In summary, the components of the expanded *pals* gene family form several regulatory modules in *C. elegans* that act as IPR ON/OFF switches and maintain the balance between immunity and development. Similar to *pals* regulators of the IPR, the antagonistic relations between different STAT proteins during IFN response activation (described earlier in this review) highlight the importance of complex and precise regulation of immunological responses.

## CONCLUSIONS

A hallmark of genes involved in host‐pathogen battles is their rapid acquisition and loss over evolutionary time, including expansion of particular gene families.^[^ ^1^ ^]^ In this light, one reasonable explanation for expansion of the *C. elegans pals* gene family is selective pressures from co‐evolving pathogens. A set of 330 *C. elegans* natural isolates has provided a powerful tool for evolutionary work in this organism, with a recent study indicating selective pressures on *pals* genes in particular.^[^ ^116^, ^117^ ^]^ There have also been examples of evolutionary expansion/loss of RLR genes over time, as RIG‐I appears to have been lost in chickens and some fish.^[^ ^118^ ^]^ RLRs may also have been lost in *D. melanogaster*, given the presence of RLRs in *C. elegans*, sea urchins and vertebrates together with the evolutionary relationships among these species.^[^ ^119^ ^]^ On the other hand, RLRs appear to have expanded independently to three genes each in *C. elegans* and mammals, and 12 genes in the purple sea urchin.^[^ ^120^ ^]^

Is the similar regulation of the *C. elegans* IPR and the mammalian IFN‐I response by RLRs and other factors mentioned above due to divergent evolution (i.e., derived from a similar pathway found in the last common ancestor of *C. elegans* and mammals), or convergent evolution (*C. elegans* and mammals independently adopted these modes of defense)? Notably, a revolution in our understanding of cell‐intrinsic immune responses regulated by NLRs has shifted the field toward the model that these immune receptors found both in plants and animals have likely undergone divergent evolution, instead of convergent evolution as originally proposed.^[^ ^121^ ^]^ These findings are based on structural similarities in animal and plant NLRs, as well as the recent finding that bacteria have NLRs used for defense. Furthermore, other cell‐intrinsic defense pathways like cGAS/STING have been shown to be deeply conserved across bacteria, archaea, and eukaryotes.^[^ ^122^, ^123^ ^]^ Thus, the last common ancestor of bacteria, plants and humans likely had versions of NLR and cGAS/STING cell‐intrinsic defense that underwent extensive sequence divergence to become their current forms that are still being used across phylogeny for anti‐viral defense.

Given the precedents mentioned in the paragraph above, together with the similarities mentioned in this review, we suggest that RLR regulation of the IPR and the IFN‐I may represent divergent evolution. As described above, although IFN genes evolved in the vertebrate lineage, several upstream activators of the IFN‐I response evolved much earlier. In particular, RLRs evolved upon multicellularity in metazoans, while other cellular processes that regulate the IPR and IFN‐I response are even more broadly conserved.^[^ ^48^, ^124^, ^125^ ^]^ While highly speculative, we suggest that surveillance of purine salvage and the proteasome may also trigger protective transcriptional responses in single‐celled organisms. If such regulation were found in single‐celled organisms, together with identification of RLRs that regulate the response, these findings would provide support for the divergent evolution model. Further analysis of anti‐viral defense responses in a broad range of hosts including *C. elegans* will inform how mechanisms of defense have evolved against intracellular pathogens in diverse hosts, including cell‐intrinsic as well as systemic signaling used to fight off infection.

## AUTHOR CONTRIBUTIONS

*Conceptualization*: Vladimir Lažetić and Emily R. Troemel. *Writing – original draft, review and editing*: Vladimir Lažetić, Lakshmi E. Batachari, Alistair B. Russell and Emily R. Troemel. *Figures*: Lakshmi E. Batachari.

## CONFLICT OF INTEREST STATEMENT

The authors declare that there is no conflict of interest.

## Data Availability

Data sharing is not applicable to this article as no new data were created or analyzed in this study.
